# Do overweight students have lower academic performance than their classmates? A pilot cross sectional study in a middle school in Tehran

**DOI:** 10.1186/s40200-014-0087-0

**Published:** 2014-08-15

**Authors:** Ramin Heshmat, Fatemeh Ardeshir Larijani, Amir Pourabbasi, Ata Pourabbasi

**Affiliations:** Chronic Diseases Research Center, Endocrinology and Metabolism Population Sciences Institute, Endocrinology and Metabolism Research Institute, Tehran University of Medical Sciences, Shari’ati Hospital, North Kargar St, Tehran, Iran; Non-Communicable Diseases Research Center (NCDRC), Endocrinology and Metabolism Research Institute, Tehran University of Medical Sciences, Tehran, Iran; Foundation for education, research and services on Educational Medicine & School Health, Tehran, Iran; Diabetes Research Center, Endocrinology and Metabolism Research Institute, Tehran University of Medical Sciences, Tehran, Iran

## Abstract

**Background:**

Recently the special attention is given to psychological aspects of childhood obesity in overweight and obese children. The present pilot study aims to investigate the association of obesity and lipid profile with school performance among Iranian students.

**Methods:**

69 middle school male students between the ages of 12 to 14 in 2009–2010 were studied. BMI was considered as the obesity index. The students’ Grade Point Average (GPA) along with their scores in math, geometry, calculus, English and absent rate were obtained as academic performance. Serum lipid profile was also assessed by a uniform method.

**Results:**

The GPA score, math and geometry scores were lower in overweight students in comparison with control group. The study of serum lipid profile indices showed no significant statistical association between serum lipid profile and school performance.

**Conclusions:**

Our results support previous findings pointing out the negative effects of obesity on students’ school performance.

## Introduction

The prevalence of obesity among the school-aged children and adolescents has considerably increased in many countries from less than 10% in Russia and East Europe to more than 30% in the U.S [1].

There exist several studies regarding the medical co-morbidities of obesity in childhood and adolescence such as increased risk of cardiovascular diseases, musculoskeletal disorders, hypertension, hypothyroidism, sleep disturbance [2–4]. It is noticeable that, recently there is a special attention to psychological aspects of childhood obesity and mental well-being in overweight and obese children [5,6]. It has been shown that body image distortion in adolescents can cause distress and depression [7]. In addition, high BMI in term of being overweight is more prevalent among 6–10 old aged psychological dissatisfied children [8]. As a result, obesity related to distress and psychological problems in children and adolescents can be accompanied with unusual behaviors for weight reduction such as strict diet or drug use [9]. One of the most challenging psychological co-morbidities of childhood obesity is poor school performance; however, certain controversy remains in this regard. Many studies emphasized on positive relationship between obesity and poor school performance [10–13], while others has proved no significant correlation between these two issues [14,15]. The majority of the existing studies have assessed the relation between Body Mass Index (BMI) and school performance without taking into account serum lipid level.

The aim of the present pilot study is to investigate the association of obesity and lipid profile with school performance among Iranian students considering the cultural diversity, ethnicity and educational characteristics in Iran.

## Material and methods

### Sampling technique

This cross-sectional study was conducted on the 69 school male students between the age of 12 to 14 in 2009–2010. They were studying in one of the private school in Tehran and they were matched socioeconomically (Table 1).

**Table 1:** **Demographic characteristics of students**

	**Variables**	**Minimum**	**Maximum**	**Mean**	**Std. deviation**
Sociodemographic	Mean age of students	12	14	13.25	0.315
	Time spent in school (Hours per day)	8.5	8.5	8.5	0
	Time spent in school (Hours per week)	51	51	51	0
	Average family income (USD per month)	1840	2970	2080.4	233.473
Growth indices	HEIGHT	134.00	170.00	151.0290	7.71929
	WEIGHT	30.00	72.00	46.6377	12.00242
	BMI	14.22	31.11	20.2310	3.98650
School performance	LANGUAGE	12.75	20.00	19.0233	1.18141
	Calculus	2.00	20.00	18.3856	2.84753
	Geometry	12.00	20.00	18.8178	1.38560
	Math	10.63	20.00	18.6017	1.86108
	GPA	15.28	19.83	18.9417	.81546
	Absent Rate	.00	11.00	1.7458	2.09756
Serum Lipid Profile	TG	34.00	241.00	84.6250	40.85242
	LDL	10.00	174.00	102.0217	29.25177
	HDL	32.00	135.00	52.2609	15.45522
	CHOL	100.00	258.00	171.4167	27.28169

None of the students had experienced chronic serious diseases or medical problems influencing their school performance based on the medical records in school.

#### Weight measurement

The anthropometric measurements including weight and height were obtained while individuals wearing light cloths and no shoes. The students' weights were measured by a single trained physician at the beginning of the school year with the same wall-mounted stadiometer and a digital scale. The BMI was calculated as dividing body weight by squared height (kg/m^2^).

Afterwards, the results were compared with the BMI age-adjusted chart for males and the students with BMI values over the 85 percentile were considered as an overweight and those with BMI values over the 95 percentile as obese ones [16,17].

#### Laboratory assessment

Early in the morning about 10 ml of venous blood samples, after 12 hours of fasting, were obtained from each student for biochemical tests. Lipid profile including triglyceride, total cholesterol, LDL cholesterol and HDL cholesterol were centrifuged for plasma concentration and they were restored at the same laboratory.

#### Evaluation of the academic performance

The students’ actual Grade Point Average (GPA) along with their score in math, geometry, calculus and English were obtained at the end of the academic year from the school records. Also the absenteeism rate was estimated for each student at the end of the educational year based on the school reports and it is considered as a one of the school performance indices.

#### Ethical approval

The project was conducted in accordance with declaration of Helsinki and the guidelines of the Iranian Ministry of Health and Medical Education. It was approved by the Ethical Board Committee of the Endocrinology and Metabolism Research Institute (EMRC). An informed consent was obtained from each participant and their parents before the investigation. No action was done without the informed consent of all participant and their parents.

### Statistical analysis

All data were analyzed using SPSS 16. A descriptive data analysis was performed to assess the subjects’ socioeconomic status, academic performance, growth indices and laboratory variables. Student’s t-distribution or its nonparametric equivalent was used when necessary. Point estimation with a confidence interval of 95% was used to compare average grades of the participants and other quantitative variables between the study groups. P-values lower than 0.05 were considered statistically significant.

## Results

A total of 69 participants with the mean age of 13.25 ± 31 years were enrolled in this pilot study. The BMI mean was 20.22 ± 3.9 kg/m^2^. In this study, 39 students were overweight and obese (BMI ≥ 85%) (Table 1).

The GPA score was lower in overweight student 18.8 ± 0.19 in comparison with the control group 19.06 ± 0.55 (p = 0.12). The same results were seen in math and geometry scores which were statistically lower in overweight students (18.04 vs. 19.1 for math and 18.44 vs. 19.15 for geometry) (p < 0.05) (Table 2). Obese students (BMI ≥ 95%) have lower grade in most subjects regarding math, geometry and English (Table 3).

**Table 2:** **Comparison of school performance indices between overweight and control group**

	**Mean (Std) in overweight (39)**	**Mean (Std) in control group (30)**	**P value**
GPA	18.8 (0.193)	19.06 (0.557)	0.12
Math	18.04 (2.456)	19.1 (0.845)	0.002
Calculus	17.64 (3.891)	19.05 (1.032)	0.4
Geometry	18.44 (1.728)	19.15 (0.881)	0.03
Language	18.89 (1.502)	19.14 (0.798)	0.31
Absent Rate*	1.6 (2.362)	1.8 (1.857)	0.15

*Day per school year.

**Table 3:** **Comparison of school performance indices between obese students and control group**

	**Mean (Std) in obese students (14)**	**Mean (Std) in control group (55)**	**P value**
GPA	18.5 (1.376)	19.05 (0.566)	0.43
Math	17.08 (3.248)	18.98 (1.043)	0.021
Calculus	16.43 (5.39)	18.88 (1.425)	0.58
Geometry	17.72 (2.309)	19.09 (0.870)	0.014
Language	18.37 (2.074)	19.18 (0.770)	0.39
Absent Rate*	1.75 (3.137)	1.74 (1.787)	0.54

*Day per school year.

Although the average grade point, in calculus and foreign language scores were lower in overweight and obese students, the differences were not statistically significant.

The absenteeism rate as an indicator of school performance was not substantially higher in overweight and obese students (p > 0.5).

The study of serum lipid profile indices obtained from 48 students showed no significant statistical association between serum lipid profile and school performance (Table 4).

**Table 4:** **Comparison of school performance indices according to serum lipid profile**

	**TG**		**CHOL**		**LDL**		**HDL**	
	**High TG group (10)**	**Normal TG group (32)**	**High CHOL group (21)**	**Normal CHOL group (21)**	**High LDL group (13)**	**Normal LDL group (29)**	**Low HDL group (3)**	**Normal HDL group (39)**
GPA	18.92 (0.83)	18.87 (0.91)	18.75 (1.11)	19.01 (0.56)	19.07 (0.76)	18.8 (0.93)	19.08 (1.12)	18.86 (0.88)
Math	18.75 (1.72)	18.54 (1.74)	18.38 (2.09)	18.8 (1.25)	19.07 (1.15)	18.37 (1.90)	18.79 (1.67)	18.58 (1.74)
Calculus	18.72 (2.20)	18.49 (2.03)	18.26 (2.37)	18.83 (1.67)	19.11 (1.28)	18.29 (2.28)	18.83 (1.25)	18.52 (2.10)
Geometry	18.77 (1.50)	18.6 (1.59)	18.5 (1.96)	18.78 (1.03)	19.03 (1.15)	18.46 (1.69)	18.75 (2.16)	18.63 (1.54)
Language	19.03 (0.92)	18.9 (1.40)	18.77 (1.73)	19.09 (0.64)	19.16 (1.04)	18.83 (1.40)	19.66 (0.47)	18.87 (1.32)
Absent Rate*	1.2 (1.39)	2.1 (2.53)	1.95 (2.74)	1.85 (1.90)	1.69 (2.01)	2 (2.49)	0.33 (0.57)	2.02 (2.38)

*Day per school year.

## Discussion and conclusion

To our knowledge, the present research is one of the first studies which demonstrated the influence of both BMI and serum lipid levels on school performance. Since prior study had run by Huang et al. in 2006 had examined the effect of both BMI and body fat index (according to foot bioelectric impedance) on academic performance [12] in which the cultural issues and ethnicity impact on the relation of obesity and school performance by student socioeconomic matching were considered.

Our results support previous findings pointing out the negative effects of obesity on school performance [10,14]. Almost all the school performance indices measured in our study were lower in obese students; however the differences were only significant for mathematics and geometry. It is noteworthy that, in this study student school performance was not in relation to serum lipid levels. Several anthropometric measurement techniques such as the triceps skin fold have been applied o determine the overweight and obese individuals [18,19]; besides, body mass index (BMI) is still the popular and accurate technique in this regard [20–22]. Although there are many methods of students’ school performance assessment such as IQ, grade rank, grade point average, days absent, visit to school nurse, enrollment in extracurricular activities, and plans to further schooling. The recent studies focused on the average grade in core academic subjects including Mathematics and English (foreign language) as a true proxy for school performance [12,23]. According to these researches GPA, math, geometry, calculus and foreign language scores and also absenteeism rate were used as a school performance in our study.

There are some investigations affirming the inverse relationship between obesity and school performance [10,14] similar to the recent systematic review and meta-analysis in 2010 which was in agreement with the correlation of high BMI and low IQ in school-aged children as a school performance proxy [24]. Furthermore, Hollar D et al. showed that the adaptation of certain anti-obesity interventions controlled not only the students’ BMI and blood pressure but also their school performance [25].

There are some potential explanation for reveres association between student grades average and BMI: firstly, as proven in previous studies there is a significant correlation between high BMI and depression [26,27], that could strongly affect student performance. Noting that, a study in North Korea on 405 students confirmed that, psychological problems in overweight and obese student are the major cause of poor school performance rather than their body image [28]. In addition, the obese students are mostly less physically activate [29,30] which lead them to experience the lower school performance compared with normal BMI students [31,32].

Beside the investigations which confirmed the negative relationship between obesity and school performance, some authors have shown the controversial results. A study run by Louisa J et al. demonstrated no association between BMI values and school performance; however, it reported a negative influence of obesity on cognitive activities [33]. Interestingly, an Italian study showed the positive relation of obesity on school performance [34].

There are two main limitations in our study: first, recording system in governmental school in Iran doesn’t have a high quality due to a large number of incomplete health reports. Hence, it is not possible to rely on the school records in researches and there should be a supervisory mechanism to control it. Moreover, this study was a pilot study in one private school in Tehran hence depriving a large sample size.

According to the existing evidences obesity has a considerable impact on students’ school performance. In line with them. Our pilot study added new evidence which confirms these findings. But the key point is that such outcomes should be adjusted for each country with variety of a large scope of life style, diet, education system, and ethnicity that probably have some influences on this relation. So that, designing observational and interventional studies with greater sample size, while controlling other influential factors are recommended to validate the relation between obesity and school performance. After confirming this, the corrective interventions might be planned according to these factors in order to manage obesity in students and subsequently improve their school performance.
